# Correction: The impact of using digitally-mediated social stories on the perceived competence and attitudes of parents and practitioners supporting children with autism

**DOI:** 10.1371/journal.pone.0270948

**Published:** 2022-06-29

**Authors:** Louis John Camilleri, Katie Maras, Mark Brosnan

There is an error in affiliation 2 for author Louis John Camilleri. The correct affiliation 2 is: Department For Inclusion & Access to Learning, University of Malta, Malta.
