# Supplementary material for: The impact of using digitally-mediated social stories on the perceived competence and attitudes of parents and practitioners supporting children with autism
Source: PLoS One. 2022 Jan 18;17(1):e0262598. doi: 10.1371/journal.pone.0262598 (PMC8765644; doi:10.1371/journal.pone.0262598)
Supplement: S1 Table — (DOCX) [file pone.0262598.s001.docx]

S1 Table: Practitioners, baseline & outcomes (n=34), descriptive statistics

|  | **Baseline** | | **Outcome** | | **Difference*** | |
| --- | --- | --- | --- | --- | --- | --- |
|  | **Mean (SD)** | **Median** | **Mean (SD)** | **Median** | **Mean (SD)** | **Median** |
| Attitude summary score – Practitioners with Extensive experience | 4.66 (0.31) | 4.67 | 4.70 (0.43) | 4.92 | 0.04 (0.44) | 0.00 |
| Attitude summary score – Practitioners with little to no experience | 4.61 (0.33) | 4.67 | 4.77 (0.28) | 4.92 | 0.17 (0.39) | 0.00 |
| Attitude summary score – Practitioners’ Total | 4.65 (0.31) | 4.67 | 4.71 (0.40) | 4.92 | 0.07 (0.42) | 0.00 |
|  | | | | | | |
| Competence summary score – Practitioners with Extensive experience | 3.95 (0.67) | 4.00 | 4.18 (0.42) | 4.17 | 0.23 (0.73) | 0.17 |
| Competence summary score – Practitioners with little to no experience | 3.46 (0.77) | 3.58 | 3.91 (0.61) | 3.92 | 0.46 (0.52) | 0.58 |
| Competence summary score – Practitioners’ Total | 3.84 (0.71) | 4.00 | 4.12 (0.47) | 4.17 | 0.28 (0.68) | 0.25 |
|  | | | | | | |
| Confidence Score – Practitioners with Extensive experience | 3.67 (0.62) | 4.00 |  | | | |
| Confidence Score – Practitioners with little to no experience | 3.33 (0.82) | 3.50 |  |  |  |  |
| Confidence Score – Practitioners’ Total | 3.61 (0.66) | 4.00 |  |  |  |  |

*SD = standard deviation*

** Difference is referring to the mean of the difference between outcome and baseline ratings.*
