# Supplementary material for: The impact of using digitally-mediated social stories on the perceived competence and attitudes of parents and practitioners supporting children with autism
Source: PLoS One. 2022 Jan 18;17(1):e0262598. doi: 10.1371/journal.pone.0262598 (PMC8765644; doi:10.1371/journal.pone.0262598)
Supplement: S2 Table — (DOCX) [file pone.0262598.s002.docx]

S2 Table: Parents, baseline & outcomes (n=14), descriptive statistics.

|  | **Baseline** | | **Outcome** | | **Difference*** | |
| --- | --- | --- | --- | --- | --- | --- |
|  | **Mean (SD)** | **Median** | **Mean (SD)** | **Median** | **Mean (SD)** | **Median** |
| Attitude summary score – Parents with Extensive experience | 4.43 (0.46) | 4.33 | 4.72 (0.35) | 4.83 | 0.30 (0.31) | 0.17 |
| Attitude summary score – Parents with little to no experience | 4.00 (0.71) | 4.00 | 4.70 (0.30) | 4.67 | 0.70 (0.58) | 0.67 |
| Attitude summary score – Parents’ Total | 4.27 (0.58) | 4.17 | 4.71 (0.32) | 4.75 | 0.44 (0.45) | 0.33 |
| Competence summary score – Parents with Extensive experience | 3.70 (0.56) | 3.67 | 3.83 (0.53) | 4.00 | 0.13 (0.42) | 0.17 |
| Competence summary score – Parents with little to no experience | 2.56 (0.38) | 2.83 | 4.43 (0.42) | 4.67 | 1.87 (0.52) | 1.83 |
| Competence summary score – Parents’ Total | 3.30 (0.75) | 3.59 | 4.05 (0.56) | 4.09 | 0.75 (0.97) | 0.33 |
|  | | | | | | |
| Confidence Score – Parents with Extensive experience | 3.50 (0.66) | 4.00 |  | | | |
| Confidence Score – Parents with little to no experience | 4.00 | 4.00 |  |  |  |  |
| Confidence Score – Parents ‘Total | 3.55 (0.64) | 4.00 |  |  |  |  |
|  | | | | | | |

*SD = standard deviation*

** Difference is referring to the mean of the difference between pre-engagement and post-engagement ratings.*
