# Supplementary material for: The impact of using digitally-mediated social stories on the perceived competence and attitudes of parents and practitioners supporting children with autism
Source: PLoS One. 2022 Jan 18;17(1):e0262598. doi: 10.1371/journal.pone.0262598 (PMC8765644; doi:10.1371/journal.pone.0262598)
Supplement: S1 Appendix — (DOCX) [file pone.0262598.s003.docx]

**Appendix A. Questionnaire Items**

| **Preliminary questions** |
| --- |
| Did you ever create/write a social story before today?  Did you ever carry out a social story intervention with a child before today? |
| **Confidence scale (CONF)** |
| CONF1 - How confident are you in your ability to write an effective social story?  CONF2 - How confident are you in your ability to effectively use a social story with a child? |
| **Attitude scale (A)** |
| A1 - I believe that a social story is an effective intervention for individuals with autism.  A2 - Using social stories is a good idea.  A3 - I consider social stories to be an adequate and appropriate intervention for children with autism.  A4 - Social stories can be used to prepare individuals with autism for a new situation they are to encounter.  A5 - Social stories can be used to help individuals with autism to decrease unwanted behaviours.  A6 - Social stories can be used to help individuals with autism learn new behaviours. |
| **Competence scale (COMP)** |
| COMP1* - I believe that social stories are difficult for me to write or create.  COMP2 - I know how to create a social story.  COMP3 - I am aware that there are specific instructions/directions on how to create a social story.  COMP4 - I am able to identify and set the goal/target of a social story.  COMP5 - I am able to structure a social story appropriately: i.e., use at least twice as many descriptive sentences as coaching sentences.  COMP6* - Social stories are tools used for entertaining children and not an intervention. |
| **User experience (UE)** |
| UE1 - Using the SOFA application was easy.  UE2 - Navigating through the SOFA application was pleasant.  UE3 - The instructions on how to use the SOFA application are clear.  UE4 - The SOFA application is user-friendly. |

**Items COMP1 and COMP6 are reverse scored.*
