# Supplementary material for: The impact of using digitally-mediated social stories on the perceived competence and attitudes of parents and practitioners supporting children with autism
Source: PLoS One. 2022 Jan 18;17(1):e0262598. doi: 10.1371/journal.pone.0262598 (PMC8765644; doi:10.1371/journal.pone.0262598)
Supplement: Attachment — Submitted filename: Response to Reviewers.docx [file pone.0262598.s005.docx]

**Response to Reviewers**

In this document we, the authors, aim to highlight the changes made in the manuscript and/or rebut some of the points raised.

- **Reviewer #5 wrote:**

The manuscript is important and well written. I hereby suggest to add the following reference (if found relevant) in introduction/review of literature section: Riga, A., Ioannidi, V., & Papayiannis, N. (2021). SOCIAL STORIES AND DIGITAL LITERACY PRACTICES FOR INCLUSIVE EDUCATION. European Journal of Special Education Research, 7(2). doi:<http://dx.doi.org/10.46827/ejse.v7i2.3773>

**Authors’ reply:**

Riga et al.’s (2021) reference is a theoretical piece which highlights how social stories (SSs), particularly digitally-mediated SSs, are useful for the autism community.

As recommended by Reviewer #5, this reference has been included in the introduction/literature review (under the subheading “Variability and the use of technology”).

We hope that this sees to all the point raised by the reviewer.

We look forward to your feedback.

Best regards

THE AUTHORS

*26^th^ December 2021*
